# Surgery for primary hyperparathyroidism

**DOI:** 10.20945/2359-3997000000557

**Published:** 2022-11-10

**Authors:** Murilo Catafesta das Neves, Rodrigo Oliveira Santos, Monique Nakayama Ohe

**Affiliations:** 1 Universidade Federal de São Paulo Cirurgia de Cabeça e Pescoço São Paulo SP Brasil Cirurgia de Cabeça e Pescoço, Universidade Federal de São Paulo (Unifesp), São Paulo, SP, Brasil; 2 Universidade Federal de São Paulo Endocrinologia e Metabolismo São Paulo SP Brasil Endocrinologia e Metabolismo, Universidade Federal de São Paulo (Unifesp), São Paulo, SP, Brasil

**Keywords:** Primary hyperparathyroidism, parathyroidectomy, treatment, parathyroid hormone

## Abstract

Primary hyperparathyroidism (PHPT) is a hypercalcemic disorder that occurs when one or more parathyroid glands produces excessive parathyroid hormone (PTH). PHPT is typically treated with surgery, and it remains the only definitive therapy, whose techniques have evolved over previous decades. Advances in preoperative localization exams and the intraoperative PTH monitoring have become the cornerstones of recent parathyroidectomy techniques, as minimally invasive techniques are appropriate for most patients. Nevertheless, these techniques, are not suitable for PHPT patients who are at risk for multiglandular disease, especially in those who present with familial forms of PHPT that require bilateral neck exploration. This manuscript also explores other conditions that warrant special consideration during surgical treatment for PHPT: normocalcemic primary hyperparathyroidism, pregnancy, reoperation for persistent or recurrent PHPT, parathyroid carcinoma, and familial and genetic forms of hyperparathyroidism.

## INTRODUCTION

Primary hyperparathyroidism (PHPT) is a hypercalcemic disorder that occurs when one or more parathyroid glands produces excessive PTH. The hallmark of this condition is elevated serum calcium accompanied by high or inappropriately normal concentrations of PTH (1). PHPT is a relatively common endocrine disease whose prevalence ranges from one to seven cases per 1,000 adults (2,3).

Surgery has remained the only definite therapy since PHPT was first described (4). Symptomatic patients and those who present with renal and/or bone manifestations are primary candidates for surgery (4). Patients younger than 50 years old and those whose biochemical indicators align with specific guidelines (4–6) should also be considered for surgery (4).

Th number of PHPT diagnoses has increased in recent years because automated biochemical screenings are now used to measure serum calcium concentration (7). This technology has also changed the clinical spectrum of PHPT from a very symptomatic disease to a less symptomatic one (8), even in developing countries (7,9).

Therefore, a safe and less time-consuming procedure with low peri-operative morbidity is needed to treat the increasing number of asymptomatic and/or oligosymptomatic PHPT patients. Indeed, surgical treatment of PHPT has transformed since the first successful parathyroidectomy performed by Felix Mandel in 1925 (10): what started as a standard bilateral neck exploration has evolved to more focal procedures. Recent parathyroidectomy techniques rely on advances in preoperative localization exams to identify abnormal parathyroid glands as well as for intraoperative PTH monitoring (11–16). This review summarizes surgical approaches for PHPT, highlights the relevance of preoperative localization studies and intraoperative PTH measurements, and discusses surgery under special conditions such normocalcemic PHPT, pregnancy, reoperation for persistent or recurrent PHPT, parathyroid carcinoma, and familial and genetic forms of hyperparathyroidism.

## ADJUVANT METHODS IN PHPT SURGICAL TREATMENT

### Localization exams

Localization workup is a set of non-invasive to invasive radiological exams that identify structural and/or functional pathological parathyroid glands. They do not confirm or exclude the diagnosis of PHPT, nor should they influence the indication for surgery (1,3). Imaging should be performed after the decision to proceed with parathyroidectomy and used only for operative planning (1,17,18). The ideal sequence of exams should be tailored according to the patient’s needs and surgeon’s preferences. Repeating negative exams adds little information and further delays surgery (19,20).

Ultrasound (US) is the most frequent localization exam whose accuracy approaches 76% (18,21) A study of 14 countries found that almost 90% of the patients underwent US and that the exam was true positive in 66.8%, misleading in 8.6%, and false negative in 22.8% of cases (22). US is the least expensive imaging modality, has no radiation, and can be performed in a medical office. It also offers valuable insights on concomitant thyroid disease (1,23). Parathyroid surgeons who are experienced in performing their own US can enhance the accuracy of adenoma identification (19,24,25). To date, the most cost-effective strategy is to combine US with other functional imaging modalities such as technetium Tc-99 m sestamibi scintigraphy (MIBI), four-dimensional tomography (4D-CT), or positron emission tomography/computed tomography (PET/CT) (1,18,23).

MIBI is the current gold standard for detecting hyperfunctioning parathyroid glands. A meta-analysis of 23 papers including 1236 patients reported a detection rate of 88% (21). MIBI and US are often combined; together their true positive rate is 58.6% for solitary adenoma. This combination was misleading in only 4.5% of cases and both negative in 8.4% (22). The major bias of large MIBI studies is the variability in image acquisition, which hinders comparisons across imaging centers. This may be why 4D-CT has garnered more attention in recent years. Surgeons are usually familiar with CT and more readily include it in the localization workup. 4D-CT is also more sensitive than MIBI (21,23,26); a study that enrolled 400 patients reported the sensitivities of 4D-CT for single gland and multiglandular disease (MGD) as 79% and 58%, respectively, against 58% and 31% for MIBI (26).

PET/CT using Methionine is typically used as a second-line imaging modality after negative MIBI (21). Its 70% sensitivity is slightly higher than that of MIBI, and its high positive predictive value (PPV) normally exceeds 95% (21). Choline is a new tracer that can be used in PET/CT to detect pathological parathyroid glands. A meta-analysis of 517 patients reported a sensitivity of 95% and PPV of 97% (27). Remarkably, this technique can accurately identify small adenomas (less than 1 cm) that conventional MIBI cannot detect; however, choline PET/CT is expensive, and inflammatory lymph nodes may absorb choline and cause false positives (18,21).

The most invasive exam is selective venous sampling for PTH dosage. is only, which is recommended only if other localization procedures are negative and in reoperations (22). Sampling both internal jugular veins can help discern on which side the hyperfunctioning parathyroid gland is located (22,28).

Negative or inconclusive imaging increases the likelihood of MGD and decreases the cure rate from 95%-97% to around 90% (20,23). Nevertheless, these results should not be an excuse for avoiding or delaying surgery (1). An experienced surgeon should consider additional localization tools or proceed to bilateral neck exploration while including the patient in the decision process (29).

Fine needle aspiration, a major tool in thyroid disease, plays a secondary role in PHPT because cytological analysis rarely adds information that localization tests have not already presented. Needle tract seeding (3,30,31), and fibrotic reactions may also hinder surgical resection and post-operative histological analysis (32). Fine needle aspiration for PHPT should be routinely avoided and reserved for exceptional cases.

### Intraoperative PTH monitoring (IO-PTH)

In 1987, Nussbaum and cols. introduced a two-site antibody technique whose sensitivity and specificity for measuring the intact PTH (1–83) molecule exceeded those of previous assays (33). In 1991, Irvin and cols. developed the rapid intraoperative PTH (IO-PTH) assay and applied it to routine clinical practice for surgical treatment of PHPT (34–37). Since 1996, rapid IO-PTH assays have become commercially available (11) and are routinely used by parathyroid surgeons (12,14–16,38,39).

Most IO-PTH assays provide results within 8-20 minutes and correlate well with standard diagnostic assays (11,39). A curative drop of IO-PTH allows the surgeon to terminate the operation and obviate additional exploration. On the other hand, failure of the IO-PTH levels to demonstrate an adequate decrement demands for further surgical exploration owing to the presumed additional hypersecreting parathyroid gland(s) presence (38). Doctors also cannot agree on which criteria of the IO-PTH decay should be used to confirm the operative cure (4,40–42); several different interpretation criteria were found to be unequal for detecting MGD and predicting cure (40–42). After excising the hyperfunctioning parathyroid tissue, most surgeons use the Miami criterion, which requires a 50% IO-PTH decay relative to the highest value of either the pre-manipulation or pre-excision sample (4,40,43). Table 1 shows other algorithms used as interpretation criteria: Vienna (44), Halle (44), and Rome (45).

**Table 1:** Various IO-PTH interpretation criteria and their accuracy in predicting post-operative serum calcium values

Criterion	Interpretation	Sensitivity (%)	Specificity (%)	PPV (%)	NPV (%)	Overall accuracy (%)
Halle (44)	IO-PTH decays into the low normal range (<35 pg/dL) within 15 min after parathyroid removal	62.9	100	100	14.2	65
Miami (35)	IO-PTH decays by 50% at 10 min post-excision of hyperfunctioning parathyroid compared to the highest of either the pre-manipulation or pre-excision sample	97.6	93.3	99.6	70	97.3
Rome (45)	IO-PTH decays by more than 50% of the highest pre-excision level, and/or IO-PTH concentration within the reference range at 20 min post-excision, and/or <7.5 pg/dL at 10 min post-excision	82.9	100	100	26.3	83.8
Vienna (44)	IO-PTH decays by 50% or more of the pre-incision value within 10 min after parathyroid resection	92.2	93.3	99.6	60.9	92.3

IO-PTH: intraoperative PTH measurement; PPV: positive predictive value; NPV: negative predictive value.

Adapted from: Barczynski and cols.; 2009 (42).

The most balanced criteria are Miami followed by Vienna (42). Yet Rome followed by Halle may be useful for the intraoperative detection of underlying MGD (11) because relying on the 50% decline as the sole IO-PTH criterion increases the rate of operative failure in patients with MGD (41). These cases warrant rigid incremental criteria such as the fall into the normal or near-normal PTH range to ensure surgical success (4,41,45,46). Merging various IO-PTH criteria tends to increase surgery cure rates (41).

Surgeons who use IO-PTH should validate their criterion to ensure operative success and to minimize excessive neck exploration (11). Our 93.4% surgical success rate with IO-PTH measurements with 91 PHPT patients revealed an average IO-PTH drop of 81.7% from the pre-incision value at 10 minutes after removing the abnormal parathyroid (38). In this study, the average IO-PTH drop was much higher than the criterion of IO-PTH >50% prescribed in the literature to ensure a cure. Additionally, the average preoperative PTH measured in those patients was 426 pg/dL, suggesting a more severe disease at our tertiary hospital. IO-PTH findings may vary across centers (38), and surgeons should evaluate IO-PTH criteria in their specific institutions to ultimately determine if neck exploration is appropriate.

Many centers have adopted minimally invasive approaches to parathyroid surgery; their cure rates exceed 98%, the same operative success rate of the classical bilateral neck exploration (4). Notably, IO-PTH does not seem to improve surgical outcomes of PHPT patients with concordant results of two preoperative imaging studies (47,48). Sartori and cols. evaluated 426 of these patients who underwent parathyroidectomy with and without intraoperative monitoring (47); IO-PTH did not benefit these patients. Barczynski and cols. concluded the same after evaluating post-operative outcomes in 177 consecutive patients with PHPT and compared the results of preoperative imaging, surgical findings, and the value-added accuracy of IO-PTH in surgical decisions (48). In both manuscripts, the cure rates among patients operated with and without IO-PTH monitoring were very similar to those in patients whose preoperative images were concordant (47,48). Therefore, surgeons should scrutinize whether IO-PTH would benefit the patient given its time and cost to perform. A reliable point-of-care device test could measure IO-PTH monitoring to reduce the durations of surgery and anesthesia (49).

During parathyroid surgery, IO-PTH assay is a valuable adjunct if focused approaches are used, obviating the need to identify normal parathyroid glands. They will not, however, replace the single most important criterion for excellent outcomes – an experienced parathyroid surgeon (4,50).

## NECK SURGERY TECHNIQUES

The only definitive treatment for PHPT is removal of all hyperfunctioning parathyroid tissue (1,3,23,51,52), which quickly normalizes calcium levels that remain stable long-term. This biochemical cure reduces the risk of nephrolithiasis and bone fracture and increases bone mineral density (3,5,53). These benefits are more apparent in patients with classical symptoms (1), suggesting that all symptomatic patients should be referred to surgery if no contraindication is presented (3,5,54). Outside of the classic target organs, other non-classical symptoms such as neurocognitive and cardiovascular may improve (1,54–56). Moreover, surgery should also be considered even for asymptomatic patients (3,52) as described by specific guidelines (4–6).

Several different techniques can be used to perform a parathyroidectomy (PTX) in the PHPT scenario. Surgery can be performed by a bilateral neck exploration (BNE), unilateral neck exploration, or a minimally invasive procedure (MIP). Prior to PTX, surgeons must gather all preoperative information and localization studies to determine the best initial surgical procedure.

BNE is the time-tested standard technique for PHPT (1). In this type of surgery, all four parathyroid glands must be identified and compared to deduce the presence of a single adenoma or MGD. It has a long-term success rate greater than 95% and few complications (1,57,58). BNE was performed in most patients in the 1990s when *Doppman* stated that “the only localizing study indicated is to localize an experienced parathyroid surgeon” (57). Yet with technological advances and more reliable preoperative imaging, less invasive techniques have displaced BNE (58): By the end of 2010, a Scandinavian study found that 61% of 2,708 patients underwent BNE (59). In 2019, a multicenter study found that only 40% of 5597 patients initially needed a BNE, and 15% needed conversion to bilateral surgery (58). An even more recent study found that a BNE was recommended as a first option to approximately 25% of patients (22). Still, surgeons rely on BNE despite its progressive decline because it can be performed regardless of preoperative imaging findings and IO-PTH dynamics (57). BNE is the best choice for patients whose preoperative images are negative or inconclusive, whose medical history suggest MGD, and those who need associated thyroid surgery (22,57). This technique is indicated primarily for more complex cases and therefore is associated with longer operative time, a higher incidence of MGD, smaller adenoma size, and a higher incidence of surgical failure than other types of surgery (22).

MIP, or focused parathyroidectomy, is a set of techniques designed to limit neck dissection only to the area of the parathyroid adenoma. MIP relies on patient’s history and laboratory data, it starts guided by positive preoperative localization exams and ends through IO-PTH decay (1,18,22). This procedure is not recommended for patients with known or suspected risk of MGD (1,18,57). Some authors believe that MIP could be performed without IO-PTH (60); this strategy is mostly adopted for patients with two positive and concordant localization exams for the same adenoma, for whom IO-PTH is not cost-effective (22). However, this remains highly debated (61). Advantageously, MIP offers shorter recovery times, a smaller incision length, reduced operative time, and a lower occurrence of post-operative complications (18,22). MIP has high surgical success rates (95%-98%) and low complication rates (1%-3%) (18).

An intermediate surgery between MIP and BNE is the unilateral approach, which is based on a positive preoperative localization exam, and during surgery, in the identification of two ipsilateral parathyroid glands (one normal and one adenoma) without the use of IO-PTH. The idea of this surgery relies on the identification of a normal gland to reduce the possibility of MGD, avoiding the need for contralateral dissection. Norman and cols. advocated for unilateral parathyroidectomy but recently published a paper on 15,000 patients, where unilateral procedures were 11-times more likely than BNE to fail, and their long-term recurrence rate approached 6%. Thus, the authors revised their position in favor of BNE, whose outstanding cure rate is 99.4% (62).

MIP has been increasingly adopted over recent years and is preferred at experienced surgical centers (54), but all parathyroid surgeons must be familiar with BNE. As Udelsman and cols. stated: “even the ideal single adenoma patient may have occult MGD” (4), conversion is always possible during MIP. Misleading preoperative exams that do not correctly identify single gland disease, IO-PTH decay failure, and reoperation are largely responsible for conversion (22,57,58,63), which can occur in up to 15% of MIP cases (3,22,58).

A new set of surgical techniques has emerged that includes endoscopic and robotic technology. Recent literature has shown that remote access is a feasible but not MIP (64,65). It is associated with more extensive dissection and higher costs than conventional open surgery (57). By far, the only real benefit of remote access is a potentially better cosmetic outcome (22).

Surgical complications such as hematoma and nerve damage are uncommon and occur in less than 1% of PHPT patients who undergo surgery (3,54,58). BNE is unsurprisingly associated with higher rates of surgical complications, readmission, and emergency department visits compared to MIP because the former is preferred for complex cases (1,22,58). There are some conflicting data related to low post-operative calcium levels; whether oral calcium and vitamin D are required at discharge is unclear. Some authors have reported higher rates of these complications following BNE (1,22), but others have found equivalent data across surgical techniques (58). Nevertheless, permanent hypoparathyroidism is a rare long-term but equally reported complication (0%-3.6%) (22).

## SPECIAL CONDITIONS

### Normocalcemic primary hyperparathyroidism

First described in 2003, normocalcemic primary hyperparathyroidism (NC-PHPT), is defined by persistently normal total and ionized calcium levels in the presence of high PTH levels after ruling out secondary causes of high PTH levels (66). This condition remains poorly characterized nearly 20 years after its first description (67); its prevalence in the literature ranges from around 0.18% (68) and 0.6% (69) up to values as high as 6.0% (70,71) and 8.9% (71,72), likely due to inherent selection bias (73).

The clinical benefits of medical and surgical interventions in patients with NC-PHPT are poorly understood (74). Surgical treatment in NC-PHPT is associated with lower long-term cure rates when compared to surgical treatment of its hypercalcemic counterpart (73). This may be attributed to the higher frequency of MGD (as high as 43.1%) (75) in patients with NC-PHPT (74–77); patients with NC-PHPT also tend to have smaller lesions than those with the hypercalcemic disease variant (73,76,78,79). MGD decreases the success of preoperative localization, increases the technical difficulty of the surgery, and requires BNE (75). Localization studies may be less likely to localize a parathyroid lesion in NC-PHPT than in patients with traditional hypercalcemic disease (80–82): the frequency of correct preoperative MIBI localization is as low as 14% in these patients (80,81). Finally, normocalcemic patients undergo reoperation more often than hypercalcemic patients (75), and high post-operative PTH levels can be expected in up to 46.5% of operated NC-PHPT patients (83).

A widely successful treatment for NC-PHPT has not yet been found (84). Specific surgical treatments cannot be recommended because of the difficulties posed by negative preoperative imaging studies, high frequency of MGD, small intraoperative parathyroid findings, and uncertainty concerning its clinical benefits.

### Pregnancy and PHPT

The incidence of PHPT in women of childbearing age is much lower, eight per 100,000 women because PHPT most commonly affects older women around 60 years old (18). An even rarer event is its occurrence during pregnancy, which represents less than 1% of all PHPT cases (18,85). Recommendations for PHPT management during pregnancy are based on limited evidence and observational studies because the condition is so uncommon.

Several adverse maternal and fetal outcomes are associated with PHPT in pregnancy. Mothers can present with nephrolithiasis, hyperemesis gravidarum, and, in severe cases, acute pancreatitis (86), pre-eclampsia, miscarriages, intrauterine growth retardation, and premature delivery (3,18,85). These complications seem to be directly related to calcium levels, especially when calcium is 1 mg/dL above the upper normal limit (18,85). Accordingly, women with PHPT who wish to become pregnant should first undergo a curative PTX (85). Calcium levels, severity of symptoms, stage of gestation, and individual risk profile determine if women with PHPT who are already pregnant should undergo PTX (3,18).

Preoperative localization exams must avoid radiation exposure; thus, ultrasound and MRI are the preferred imaging modalities (18,85). Surgery can be safely performed in the second trimester (3), and it should be a MIP guided by localization exams and IO-PTH (18). Fetal mortality in medically treated pregnant women was estimated to be one in five fetuses (16%), while fetal mortality and morbidity in those who were treated surgically for PHPT were estimated to be 3% and 10%, respectively, according to case reports from 1930 to 1990 (86). Older literature is believed to represent more severe cases of PHPT in pregnancy with worse outcomes (87). Early recognition of PHPT with a milder degree of hypercalcemia has been associated with lower rates of adverse fetal and neonatal outcomes (86).

### Reoperation for persistent or recurrent PHPT

The first six months after PTX is a critical period for distinguishing between persistence and recurrence. Persistence is the presence of hypercalcemia before the six-month post-operative period ends. Recurrence is the evidence of hypercalcemia in patients successfully treated with previously documented normocalcemia after the six-month period. Their prevalence is highly variable in the literature; reports range from 1% to 9.8% (22,58,85).

Regardless of time and classification, the diagnostic and preoperative exams must be scrutinized after parathyroid surgery for every patient with hypercalcemia. Intraoperative information and pathological reports must be considered. Family history may help elucidate the diagnosis, especially if first-degree relatives have had hypercalcemia (3,85).

Importantly, the surgeon’s inability to find the abnormal parathyroid is responsible for approximately two-thirds of all failed operations; missed MGD accounts for the remainder of cases (85,88). Operative failure is most likely to occur in patients who previously underwent anterior neck surgery (thyroid or parathyroid surgeries), whose localization exams were non-concordant, negative, or misleading, who only performed one localization exam prior to surgery, who did not used IO-PTH or whose decay was insufficient, or whose PHPT surgery was converted from MIP to BNE (3,85,88,89).

A new set of localization exams must be performed after recurrence or persistence is established. Surgery should only be proposed following a combination of positive localization exams (3), and the decision to perform a reoperation must be tailored to each patient, since redo surgery have a whole different cost-benefit ratio. Dissection through scar tissue enhances the odds of parathyroid devascularization and recurrent laryngeal nerve injury, which substantially increase the risk of post-operative transient and permanent hypoparathyroidism and vocal fold paralysis (85). This is especially true in patients with mild disease and/or severe comorbidities, for whom medical management with cinacalcet and bone-protecting agents can be considered as an alternative to reoperation (85).

If the odds are in favor of a successful surgery, patients should be referred to a high-volume center or to an experienced parathyroid surgeon. Preoperative localization exams should be performed more comprehensively, and the use of IO-PTH is strongly advised (1,3,85). A BNE should be performed if MGD is suspected.

### Parathyroid carcinoma

Parathyroid carcinoma (PC) is a rare endocrine neoplasm that accounts for less than 1% of all cases of PHPT and affects men and women equally in their mid-40s or 50s (90). PC is typically a sporadic disease but can also be a part of a genetic syndrome, particularly of hyperparathyroidism-jaw tumor syndrome in which up to 15% of patients develop a PC (91). There is no evidence of malignant transformation from a preexisting adenoma (90,91), and radiotherapy to the neck and end-stage renal disease are the only known risk factors (92).

A presumptive preoperative diagnosis is crucial for successful management of PC, and this is possible through careful observation of its signs and symptoms. Up to 90% of patients with PC present target organ symptoms, especially renal and skeletal involvement. They may also show signs of severe hypercalcemia such as polyuria or polydipsia, myalgia or arthralgia, weakness, fatigue, depression, pancreatitis, or weight loss (90,92). Some may even experience hypercalcemic crisis, a life-threatening condition during which the cardiac, gastrointestinal, renal, and central nervous systems rapidly deteriorate (93). PC typically grows slowly, invades locally, and features metastatic dissemination. A painless, palpable anterior neck mass is the most frequently (if not the only) reported physical abnormality (90). Laboratory tests usually show very high calcium levels (Ca > 14 mg/dL or 3.0 mmol/L), high PTH (>2× upper limit of normal range), and a single large gland identified on localization exams (90–92,94).

Surgery is the first-line therapy for PC and final opportunity to perform a presumptive diagnosis and therefore to adjust the surgical extension. An intraoperative observation of a firm, adherent tumor likely indicates PC (90), after which an en-bloc resection should be performed. This procedure excises the enlarged parathyroid and any structures attached to it. Notably, prophylactic resection of unaffected cervical structures does not improve survival (91).

The histological definition of PC is complex; invasion of adjacent tissues and metastases are the only definitive criteria (90). Most parathyroid lesions lack these characteristics. Even reportedly benign lesions that presumably indicate PC should be monitored rigorously.

Recurrence rates above 50% (90–92) have been reported and include the following risk factors: less than en-bloc resection, metastatic disease at presentation, positive final pathological margin, and a final pathology report of PC in a patient with no presumptive preoperative diagnosis (92). Whenever possible, surgical resection of functional lesions and/or tumor debulking is the best option to treat relapse (92).

A large retrospective cohort of 885 patients with PC found that radiotherapy did not improve survival and should only be considered for patients who are not candidates for reoperation (95). Scarcer data on the use of chemotherapy are also not promising (92). The five- and ten-year survival rates are approximately 85% and 50%, respectively, for all patients with PC (92,95). Mortality is generally due to intractable hypercalcemia rather than tumor burden.

### Familial and genetic forms of hyperparathyroidism

Multiple endocrine neoplasia type 1 (MEN1) is an autosomal dominant hereditary syndrome with high penetrance; the mutated tumor suppressor gene *MEN1* (chromosome 11q13) causes tumors to form in the endocrine glands. Over 80% of cases are inherited forms, and the remainder are new mutations (96). Classic features include parathyroid adenomas, duodenopancreatic neuroendocrine tumors, and anterior pituitary adenomas (known as the three Ps) (97). PHPT is the most common and earliest manifestation in MEN1 with a 100% penetrance by age 50. In comparison to sporadic cases, hypercalcemia typically occurs at an earlier age (around ages 20-25), has no female predominance (equal male to female ratio), and ultimately involves all four glands in all patients (96,98). Patients usually have mild hypercalcemia, but kidney (urolithiasis and chronic kidney disease) and bone (low bone mineral density) symptoms are common (96,97).

Surgery is the treatment of choice, although its optimal timing and type are debated (98). Like other forms of familial PHPT, surgery to treat MEN1 should aim to achieve normocalcemia for as long as possible, mitigate definitive hypoparathyroidism, and facilitate potential reoperations (97). Thus, surgical treatment should be tailored to each patient.

The first surgical treatment can be delayed in mild and asymptomatic young patients to avoid symptomatic hypoparathyroidism and to reduce the number of total surgeries over their lifetime (97,98). Nevertheless, some authors support early intervention because the effects of mild PHPT on peak bone mineral density are still unknown (97).

The preferred surgical approach is a BNE with identification of all four glands. Subtotal (fewer than four) or total parathyroidectomy with immediate autograft is commonly performed. A subtotal approach has a lower incidence of post-operative hypoparathyroidism but higher rates of persistence and/or recurrence, which require a second neck exploration. Alternatively, total parathyroidectomy with autograft has a lower recurrence rate but higher incidences of subsequent hypoparathyroidism (98). A stepwise approach of removing only enlarged glands (99) or a unilateral clearance (97,100) guided by preoperative localization imaging is a potential surgical approach because MEN1 can present as only asynchronous parathyroid adenomas. To decrease recurrence rates, many authors recommend bilateral cervical thymectomy to clear embryogenic parathyroids nest found in the thymus.

Unfortunately, approximately two-thirds of patients with MEN1 die from related syndromic tumors, especially duodenopancreatic and thymic neuroendocrine malignant tumors (96). Although the thymic carcinoid tumor is a rare presentation, bilateral cervical thymectomy can help mitigate the occurrence of thymic tumors; complete thymus excision, however, is not possible with a cervical approach (96,100).

Multiple endocrine neoplasia type 2A (MEN2A) is an inherited disorder related to germline mutations in the RET proto-oncogene (chromosome 10q11). Its hallmark is medullary thyroid carcinoma, which occurs in most patients with a RET mutation (95%-100%). The MEN2A phenotype also includes pheocromocytoma, PHPT, and other non-neoplastic manifestations (101), whose aggressiveness and incidence are related to distinct subtypes of RET mutations. In the set of PHPT-related to MEN2A, most cases occur in the presence of the 634-codon mutation whose incidence is approximately 30% of patients. Other mutations such as 611, 618, 620, and 630 have an incidence of 10%, and some patients with mutations never manifest PHPT (96,101). Understanding how phenotype correlates with genotype is essential for optimizing the treatment.

The MEN2A-related form of PHPT is mild; younger patients are typically asymptomatic (101). Its low penetrance is usually associated with only one or two enlarged glands. Thus, surgeons prefer to remove only the pathological parathyroid glands, which offer positive short- and long-term outcomes (101).

The treatment of MEN2A is difficult because it includes two conditions: medullary thyroid carcinoma and PHPT, which are not necessarily synchronous, despite good outcomes reported by some centers after treating both simultaneously during the initial surgery (101). Indeed, many patients will require a total thyroidectomy years before PHPT develops, and reoperation of the central compartment is always challenging. Therefore, it is crucial to use risk stratification, perform a complete preoperative workup, and macroscopically analyze the parathyroid glands during the initial surgery. Patients with RET mutations at high risk of PHPT should have their calcium levels annually checked (101).

Finally, hyperparathyroidism-jaw tumor syndrome (HPT-JT) is a rare hereditary autosomal dominant disorder with variable and incomplete penetrance (3). Its hallmark is PHPT associated with ossifying fibroma of the maxilla and/or mandible as well as uterine and renal tumors. The PHPT can be attributed to single or MGD, and HPT-JT patients have a 15%–20% risk of developing PC (3). Treatment be based on the clinical presentation of single or MGD as previously described, and an en-bloc resection is recommended if PC is suspected (3).

## FINAL COMMENTS

Here, we summarized various aspects of surgical treatments for PHPT, ranging from MIP and its required tools to surgery for PHPT during special conditions. Although PHPT has recently become a common and less symptomatic disease, its surgical treatment still challenges the medical community; surgeons’ debate over the optimal surgical procedure to treat mild PHPT or exceptional cases of PHPT. Surgical treatment of PHPT is nuanced and motivates further studies to better treat patients.
